# Lead toxicity and genetics in Flint, MI

**DOI:** 10.1038/npjgenmed.2016.18

**Published:** 2016-06-22

**Authors:** Jacquelyn Y Taylor, Michelle L Wright, David Housman

**Affiliations:** 1 School of Nursing, Division of Primary Care, Yale University, Orange, CT, USA; 2 Department of Biology, Massachusetts Institute of Technology, Cambridge, MA, USA

It has been well established that lead poisoning, as defined by the CDC as blood lead levels (BLLs) at or above 5 μg/dl, can lead to long-term neurotoxic effects in children and requires immediate treatment.^1,2^ As such, the CDC has long recommended clinicians’ assess to all patients for lead exposure and test BLLs for all at-risk patients.^1^ Furthermore, it is increasingly recognised that there is no safe level of lead for children due to the irreversible lifelong detrimental effects of lead exposure.^2,3^ Since the disaster of lead contaminated drinking water in Flint, MI has been uncovered, action has been taken to test children for lead poisoning. However, when children are tested and results show that lead levels are below the 5 μg/dl criteria no further follow-up is conducted with these children, as they are deemed ‘healthy’. This practice is problematic, given that other studies have shown that blood lead levels, even at rates lower than the poison range can be detrimental to a child’s health.^3–5^ The estimated population of Flint is ~99,002, with about 27% of the residents categorised as children under the age of 18 years.^6^ Therefore, more than ~26,730 children, of whom 60% are African Americans (*N*=16,038), have been exposed to environmental lead in the drinking water.

A key factor, which almost certainly affects the range of susceptibility to lead poisoning, is a child’s genetic makeup. Current models for the neurotoxic effects of lead implicate the enzyme arylsulfatase A (ASA) as a particularly significant target of lead in the central nervous system (CNS).^7^ Reduced levels of cellular ASA by lead has been suggested to augment the other detrimental affects of the metal, resulting in the death or impaired function of oligodendroglia progenitor cells (OPCs) and lead to CNS dysfunction. Certain single-nucleotide polymorphisms (SNPs) of the gene for ASA (*ARSA*) cause greatly reduced levels of the enzyme with no obvious phenotype. One of these (Asn_350_Ser), first characterised in 1989,^8^ when homozygous, causes up to a 60% reduction in the intracellular levels of the enzyme.^9^ The homozygous presentation results in metachromatic leukodystrophy, leading to loss of developmental milestones in children and death at a young age. Low ASA activity in individuals with a heterozygous presentation can be identified via signs and symptoms, and it is suggested that these symptoms may be amplified when the individual is exposed to even low levels of environmental lead.^4^ Some symptoms of this heterozygous pseudodeficiency and environmental lead exposure may include: learning disabilities; behaviour problems' high blood pressure; tremors; seizure disorder; low sperm count and so on. This SNP is of particular relevance to the current situation in Flint, MI because a study conducted by one of us (J.Y.T.) and colleagues in 2002^4^ of 107 African-American children in Detroit showed that this population had a gene frequency of ~0.45 for the Asn_350_Ser SNP, heterozygosity at this position often referred to as a pseudodeficiency. This frequency is much higher than what is seen in people of European ancestry (CEU=0.14) and higher frequency of this allele has been consistently reported in populations of African ancestry (ASW=0.36).^10^ These findings suggest that the Flint, MI population suffering lead exposure requires a more effective approach than simply measuring lead levels and setting a cutoff at 5 μg/dl. The question of the appropriate response to the interaction of genetics and lead toxicity was recently commented on by Poretz^7^ who stated ‘Identification of susceptible children for targeted concern and treatment would help alleviate the impact of the toxicant on the at-risk population.’ We agree strongly with this premise. Genotyping, followed by targeted intervention in Flint, of children who are at higher risk for lead poisoning should be carried out immediately, particularly for those who test below the poison mark. The current cutoff for clinical intervention at 5 μg/dl is inadequate and incorrect, especially for children who are carriers or homozygous for the Asn_350_Ser SNP.

On the basis of these works, it is strongly suggested that children in Flint be genetically tested for ASA pseudodeficiency (heterozygotes) and risk assessed for neurological deficits due to long-term environmental lead exposure. Currently, this testing is not being conducted, which means people with ASA carrier status may experience detrimental effects at a lower exposure threshold, will not receive much-needed treatment that would help mitigate long-term health problems. Not treating people, particularly children, who may be at risk of both ASA pseudodeficiency with concurrent lead exposure could lead to long-term health problems including neurological impairment, neuropsychiatric disorders, increases in blood pressure and more. On the basis of this hypothesis, there is no time to waste testing and caring for lead-exposed people, particularly children, as this is an urgent matter that further exemplifies the need for immediate action.

Because such a large population in Flint has been affected by environmental lead exposure, this would be the prime sample for examining the mechanistic biochemical pathways involved in expression of symptoms and for changes in clinical care protocols among an effected sample not controlled by laboratory conditions. Flint offers a unique environmental factor where all of the residents have been exposed to environmental lead in the drinking water, which is why all children should be tested. We believe that all children should be tested for all possible genotypes of the ASA pseudodeficiency.

If these children are shown to be carriers, then the families should be afforded the same opportunities for treatment that children with high level of lead exposure receive (e.g., nutritional interventions to decrease absorption, physical therapy, behavioural therapy, educational assistance and so on). If these children are not tested and treated, this is a gross missed opportunity for nurses and other health providers to utilise precision medicine techniques to its’ full potential to help improve the health of people in need. Ignoring symptoms, their underlying causes, and deeming patients as healthy with no requirement for follow-up goes against the premise of ‘symptom science’ detailed by the National Institutes of Health/National Institute of Nursing Research/ and we as health providers can and will do better. Now is the time to use symptom science in combination with genomics to improve lives and healthcare of an otherwise underserved population in the United States. Families can be easily tested with non-invasive measures such as saliva samples. The risk (cost)-benefit ratio of genotyping consenting individuals in Flint lies in favour of such testing. Such genetic testing may well lead to improvement of the physical, physiological and/or mental health of those exposed to the lead contaminated drinking water. This is an initial suggestion that requires high priority in terms of immediate and long-term follow-up of people exposed long-term to the environmental toxin lead.

Information gathered by using a precision approach in Flint can be used in other metropolitan areas and the approach can be applied to investigate variation in symptoms when large-scale toxic exposures occur. Flint is not the first community to experience lead toxicity from the water supply, in the early 2000s a similar exposure occurred in Washington DC in a predominately African-American quadrant of the city. As cities continue to age, infrastructure deteriorates and debt burdens increase, there remains potential for similar catastrophic events to occur. The type of precision genetic-testing approach proposed here can be utilised not only in Flint but also in other at-risk communities in the United States and Globally.
